# OTEH-5. Characterization of long-non coding RNA associated ceRNA network hub gene involved in glioblastoma multiforme lipid metabolism

**DOI:** 10.1093/noajnl/vdab070.044

**Published:** 2021-07-05

**Authors:** Fatin Nabihah Rizalhanafi, Norlisah Ramli, Vairavan Narayanan, Khairunnisa Rashid, Jesminder Kaur Singh, Kamariah Ibrahim

**Affiliations:** 1 Department of Biomedical Science, Faculty of Medicine, University of Malaya, Kuala Lumpur, Malaysia; 2 Department of Biomedical Imaging, Faculty of Medicine, University of Malaya, Kuala Lumpur, Malaysia; 3 Department of Surgery, Faculty of Medicine, University of Malaya, Kuala Lumpur, Malaysia

## Abstract

**Background:**

Glioblastoma multiforme (GBM) are the major death contributor in primary brain tumour. Despite having an improved diagnostic criterion by integrating both histological and molecular features such as Isocitrate Dehydrogenase (IDH) detection, the prognosis of GBM patients still remain poor. Lipid metabolism is an essential pathway that fuel GBM aggressiveness. IDH1 one of the key enzyme that regulates it. Long non-coding RNAs (lncRNAs) act as competing endogenous RNAs (ceRNAs) in tumour initiation and progression. In parallel, miRNA-mediate ceRNA crosstalk between lncRNAs and mRNAs. In this study, we aim to investigate the IDH1 subgroup lncRNA associated ceRNA network hub gene responsible in the coordination of glioblastoma multiforme lipid metabolism using bioinformatics approach.

**Methods:**

TCGA-GBM dataset consist of 168 GBM RNA-seq (159 IDH1 wt and 9 IDH1 mutation) were downloaded. Differentially expressed genes (DEG) were then obtained using Limma. Gene sets related with lipid metabolism from GSEA-MSigDB were overlapped with DEG using Venn diagram to identify the DEmRNA that are related with lipid metabolism. Construction of mRNA-miRNA and lncRNA-miRNA interaction networks were performed using miRNet. The ceRNA interaction network were later combined in the Cytoscape software. Potential lncRNA hub genes were identified by CytoHubba analysis.

**Results:**

From 1389 DEG, 67 genes were identified to be significant in the regulation of lipid metabolism. By analysing the lncRNA-miRNA-mRNA interaction network, candidate hub lncRNAs consists of three genes with highest connective nodes; CYTOR, LOXL1-AS1 and HOTAIR. These genes are significantly upregulated in glioma. LOXL1-AS1 serve as an excellent prognostic biomarker for both glioma and glioblastoma as the effect of high and low LOXL1-AS1 expression on patients’ survival is significant (p<0.05).

**Conclusions:**

Data mining and bioinformatics approach guided the identification of the potential hub lncRNAs associated ceRNA network in GBM lipid metabolism. This allows us to uncover the novel role of lncRNA in GBM tumorigenesis.

